# Case report: PET/CT, a cautionary tale

**DOI:** 10.1186/1471-2407-7-147

**Published:** 2007-08-03

**Authors:** Jayson Wang, Gary Cook, John Frank, Roberto Dina, Naomi Livni, John Lynn, William Fleming, Michael J Seckl

**Affiliations:** 1 Department of Medical Oncology, Imperial College School of Medicine, Charing Cross Hospital, Fulham Palace Road, London, UK; 2 Department of Nuclear Medicine, Royal Marsden Hospital, Downs Road, Sutton, UK; 3 Department of Radiology, Imperial College School of Medicine, Charing Cross Hospital, Fulham Palace Road, London, UK; 4 Department of Pathology, Imperial College School of Medicine, Charing Cross Hospital, Fulham Palace Road, London, UK; 5 Department of Surgery, Imperial College School of Medicine, Hammersmith Hospital, Du Cane Road, London, UK

## Abstract

**Background:**

The use of combined positron emission tomography/computerised tomography (PET/CT) scanners in oncology has been shown to improve the staging of tumours and the detection of relapses. However, mis-registration errors are increasingly recognised to be a common pitfall of PET/CT studies.

**Case Presentation:**

We report a patient with a germ cell tumour of the testis, who underwent a PET/CT scan to detect the site of relapse with a view to surgical removal. However, the PET/CT scan mislocalised the tumour site to be within the T2 vertebral body. A subsequent endoscopic ultrasound scan however showed the tumour to be anterior to the vertebral body, which was confirmed at surgery.

**Conclusion:**

In this report, we highlight the artefactual mislocalisation errors which may occur with PET/CT imaging, and the need to review and verify these scans.

## Background

Positron emission tomography (PET) scanning using 2-^18^F-fluoro-deoxy-D-glucose (^18^FDG) uptake has been in clinical use for over a decade [1,2]. The advantage of PET scanning is that it provides functional information of lesions detected, and can help distinguish between malignant and non-malignant tissues. However, PET scanning has poor spatial resolution in terms of localising lesions. In contrast, computerised tomography (CT) scanning give good anatomical, but not functional information. Since 2000, purpose-built combined positron emission tomography/computerised tomography (PET/CT) scanners have been in use which overcome the respective shortcomings of PET and CT scans [3,4]. The use of PET/CT scans therefore holds much promise in the advancement of tumour localisation and management. Nevertheless, several pitfalls remain [5]. Of these, the biggest problem is that of accurate image alignment. This case report illustrates an example of mislocation of a tumour in a patient with germ cell tumour (GCT) by PET/CT, and the subsequent potential clinical impact which results.

## Case presentation

A 38-year old man presented in 1988 with a left testicular mass which was removed and found to be a non-seminomatous GCT. Staging investigations revealed spread to the retroperitoneal lymph nodes. Six cycles of bleomycin, cisplatin and etoposide (BEP) chemotherapy was initially given [6], followed by retroperitoneal lymph node dissection (RPLND) for residual disease. In 1993, his alpha-fetoprotein (AFP) level began to rise. A radiolabelled anti-AFP-antibody scan suggested active disease in the right para-aortic region corresponding to a node near the right renal vein on CT. This was subsequently resected. Post-operatively, the AFP initially fell but then increased and the patient received six cycles of cisplatin, vincristine, methotrexate, bleomycin alternating with actinomycin D, cyclophosphomide and etoposide (POMB/ACE) chemotherapy [7]. In 1995, the patient had a further relapse with an AFP rise, and a CT scan showed recurrence in the abdomen. Following further tumour resection the patient underwent high dose chemotherapy (with carboplatin, cyclophosphomide and etoposide) with autologous stem cell rescue. In 1997, the patient's AFP level increased again. Serial CT and 18-fluoro-deoxyglucose-positron emission tomography (^18^FDG-PET) did not initially reveal any new suspicious lesions. However, when the AFP rose to 2494 ng/ml in 2001, an ^18^FDG-PET scan demonstrated a lesion in the left superior mediastinum which matched a 1.4 cm lymph node on CT. This was resected, and histology showed active yolk sac tumour involving the excision margins of the lymph node. Consequently in May 2001 he received four cycles of gemcitabine, carboplatin and paclitaxel followed by a second high dose chemotherapy procedure modified to include paclitaxel (carboplatin, cyclophophamide, etoposide and paclitaxel) [8].

**Figure 1:**
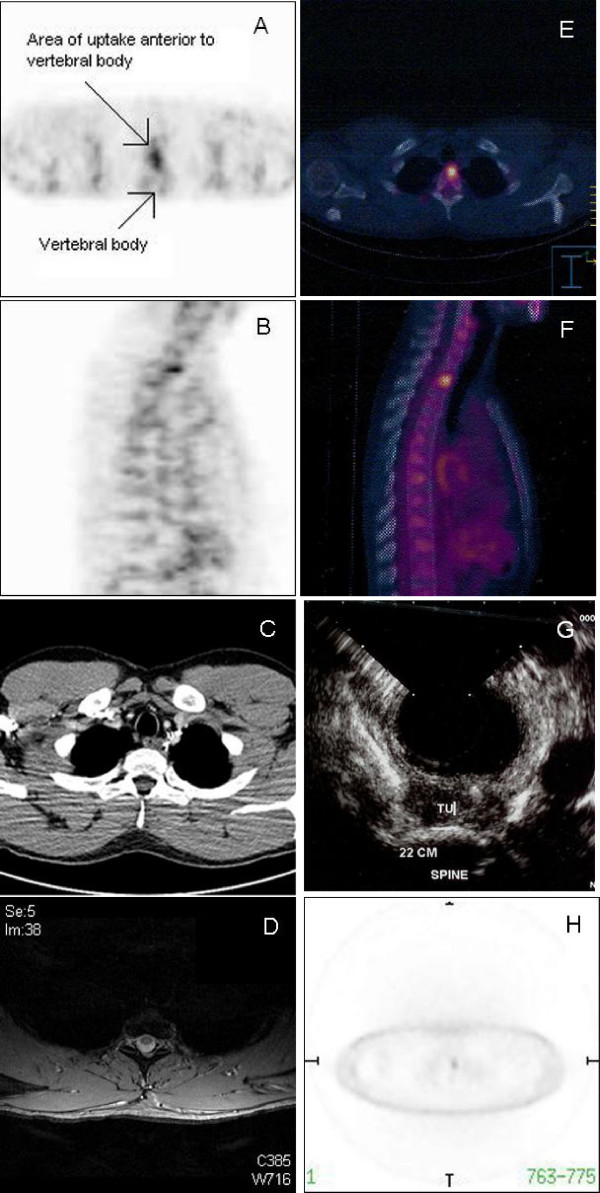
A & B: Axial and sagital view of ^18^FDG-PET scan of the patient showing region of ^18^FDG uptake anterior to the vertebral body. C: CT scan of the positive region identified on the ^18^FDG-PET scan. D: MRI of the thoracic spine in the region identified on the PET scan. E & F: Axial and sagital view of PET/CT scan of the patient showing that the lesion with ^18^FDG uptake was within the vertebral body. G: Endoscopic ultrasound image showing the tumour anterior to the vertebral body. H: Non-attenuated PET scan image from the PET/CT scan.

**Figure 2:**
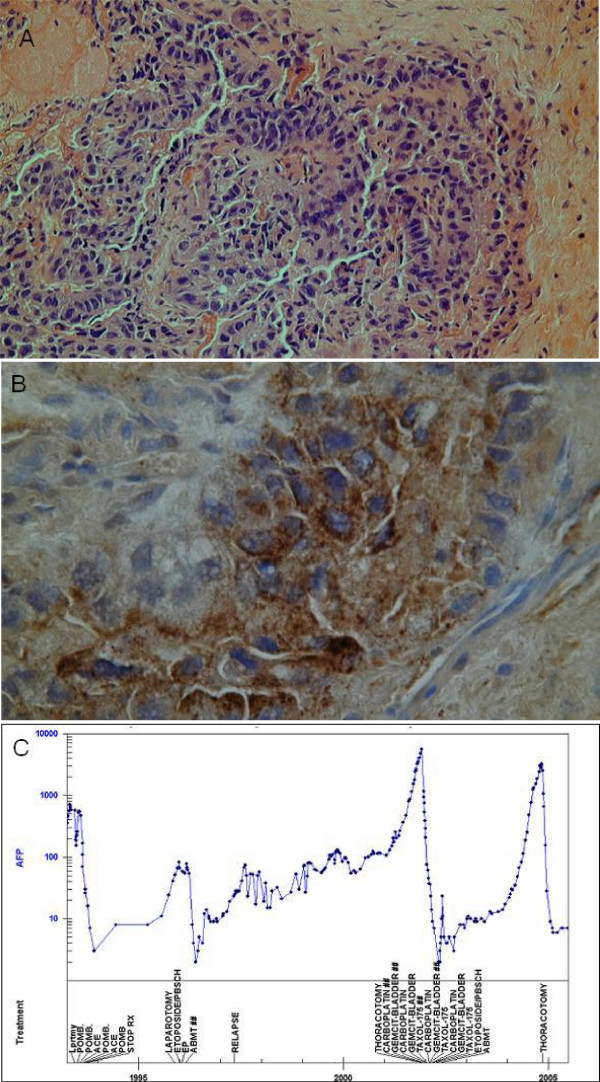
A: Haematoxylin and eosin stained section of the tumour resected from the mediastinal of the patient (100× magnification). The tumour nodule showed evidence of organoid arrangement. B: Section of the resected tumour showing tumour cells expressing AFP, as detected by immunohistochemistry (600× magnification). C: Graph of the AFP marker profile from 1993 to 2005 showing the history for relapses and treatments received by the patient.

In January 2004, the AFP level began to rise again. Extensive investigations including a CT of the chest, abdomen and pelvis, ^18^FDG-PET scan, magnetic resonance imaging (MRI) of the brain, microbubble ultrasound of the liver, and ultrasound of the remaining testis did not reveal any sites of relapse. Eventually, when the AFP measured 1531 ng/ml in Sep 2004, a repeat ^18^FDG-PET scan showed an area of increased uptake in the posterior aspect of the superior mediastinum just anterior to the vertebral bodies of T1/2 (Figure 1A and 1B). However, a repeat contrast-enhanced CT scan and MRI again showed no obvious defined lesion could be identified in this area, although there was soft tissue changes related to the previous surgery in the area (Figure 1C and 1D). The MRI scan, in particular, did not show any abnormal marrow signals. To further delineate the lesion, the patient underwent a PET/CT scan. This indicated that the lesion lay in the anterior part of the vertebral body of T2 (Figure 1E and 1F). Because of the discrepancy between the PET/CT and the initial PET scan, he then underwent a trans-oesophageal endoscopic ultrasound. This showed a 1.3 cm lesion posterior to the oesophagus and anterior to the vertebrae at approximately T1/2 (Figure 1G). At surgery in November 2004, a retro-oesophageal pre-vertebral tumour was found (1.9 × 1.3 × 0.4 cm) which was resected. Histological examination with immunohistochemical staining was consistent with relapsed GCT composed of yolk sac elements (Figure 2A and 2B). Following surgery, the patient made a good recovery and his AFP normalised in January 2005 (Figure 2C). He remains well with normal markers on close follow-up.

## Conclusion

The case presented here illustrates several learning points. Firstly, tumour markers surveillance remains a central part of the early detection system for identifying patients with relapsing GCTs. Indeed, a rising AFP preceded detection of the site of recurrence by 5 years and by 10 months in the 2001 and 2004 relapses, respectively. Secondly, if all other imaging is negative, ^18^FDG-PET scanning can actually be the first imaging modality to pick up the site of relapse, as ^18^FDG-PET scanning can be superior in terms of sensitivity and specificity to CT in detecting sites of cancer [9,10]. Thirdly, the PET/CT scan misplaced the lesion within the body of T2. An endoscopic ultrasound resolved the issue confirming the location as just posterior to the oesophagus and anterior to the vertebral body. In the absence of the latter information, the patient would have undergone much more extensive surgery including resection of the body of T2.

So why did the PET/CT mislocate the lesion? The most likely possibility is misregistration of the CT and PET images. In particular, there may be movement artefact due to respiration effects, which is increasingly recognised [5]. CT acquires imaging data rapidly within one breath-hold, whilst PET takes many minutes to accumulate a composite image during which time the patient is breathing. Consequently, peripheral lung lesions maybe mislocated by 15 mm on PET when compared to CT [11]. In our patient, misregistration due to breathing could have occurred, but given the central location of the lesion (that is, not in the lung periphery) this seems less likely. Furthermore, recent PET/CT scans use respiration-averaged CT to match PET images or respiratory gating of the PET acquisition to improve on misregistration issues [12]. This is further improved by more detector rows in the scanner [13].

Since the PET and CT scans are performed consecutively, any patient movement between the two scans will potentially also lead to misregistration. Therefore, the isolated PET and CT images from the PET/CT scan in our patient were reviewed by the reporters (who are qualified in both radiology and nuclear medicine) as well as in our multidisciplinary meetings with radiology input. The isolated PET images from the PET/CT was equivocal for whether the lesion was in the vertebral body or anterior to it (Figure 1H), while the CT scan (which was without contrast) was not able to localise the tumour. Although there was no obvious misalignment of the skin surface or other anatomical markers between the two scans, it was most likely that a minimal degree of patient movement between the PET and CT scanning (as little as 0.5 cm in this case) was sufficient for the lesion to appear to be in the vertebra rather than just anterior to it.

In summary, although PET/CT scanning holds much promise in the advancement of tumour localisation, our case report demonstrates a potential danger in relying solely on PET/CT scanning for the management of this patient. This is further highlighted in a recent study which showed that misalignment between CT and PET data can occur from 2% to up to 50% of PET/CT scans [12,14]. While these issues are becoming well-recognised in radiology, the potential for PET/CT to mislocalise lesions is not well appreciated by clinicians requesting these scans. We therefore suggest that in cases such as this, where there is a discrepancy between in the PET and PET/CT, a disclaimer ought to be inserted in the PET/CT report to highlight the potential misregistration which may occur. Furthermore, what has not been addressed is the clinical impact that these misregistrations may have. This report underlines the importance of reviewing PET/CT images and obtaining confirmatory/complementary anatomical imaging of ^18^FDG-PET-defined lesions prior to embarking on major surgery.

## Abbreviations

^18^FDG – 2-^18^F-fluoro-deoxy-D-glucose

AFP – alpha-fetoprotein

BEP – bleomycin, cisplatin and etoposide

CT – computerised tomography

MRI – magnetic resonance imaging

PET – positron emission tomography

POMB/ACE – cisplatin, vincristine, methotrexate, bleomycin alternating with actinomycin D, cyclophosphomide and etoposide

RPLND – retroperitoneal lymph node dissection

## Competing interests

The author(s) declare that they have no competing interests.

## Authors' contributions

Jayson Wang and Michael J Seckl are the main authors; Gary Cook is a co-author and provided images/figures; John Frank provided images/figures; Roberto Dina provided images/figures; Naomi Livni, provided images/figures; John Lynn was involved in patient management; William Fleming was involved in patient management; Michael J Seckl is the corresponding author.

## Pre-publication history

The pre-publication history for this paper can be accessed here:


